# Comparison of the rates of emergent otologic adverse events following mRNA COVID-19 versus influenza vaccination: a matched cohort analysis

**DOI:** 10.3389/fneur.2025.1637870

**Published:** 2025-08-07

**Authors:** Tina Munjal, Shelley Batts, Saurabh Gombar, Konstantina M. Stankovic

**Affiliations:** ^1^Department of Otolaryngology – Head and Neck Surgery, Stanford University School of Medicine, Stanford, CA, United States; ^2^Department of Otolaryngology, Head and Neck Surgery, Massachusetts Eye and Ear, Boston, MA, United States; ^3^Department of Pathology, Stanford University School of Medicine, Stanford, CA, United States; ^4^Atropos Health, New York, NY, United States; ^5^Department of Neurosurgery, Stanford University School of Medicine, Stanford, CA, United States; ^6^Wu Tsai Neurosciences Institute, Stanford University, Stanford, CA, United States

**Keywords:** COVID-19, vaccination, influenza, hearing loss, tinnitus, vertigo, aural fullness, otalgia

## Abstract

**Background:**

Otologic adverse events (AEs) have been occasionally reported as sequalae of COVID-19 vaccination, although their incidence in comparison with that of preexisting vaccines with high uptake remains unclear. This study compared the rates of new-onset otologic AEs among matched adults receiving mRNA COVID-19 vaccination versus influenza vaccination.

**Methods:**

This retrospective cohort study used electronic health records (EHR) data from Stanford Health Care to identify adults aged 50–89 years with no history of otologic disorders prior to first Pfizer/Moderna COVID-19 vaccine (December 2020–January 2022) or any pre-pandemic influenza vaccine (January 2016–December 2019). Patients were categorized by vaccination into FluVax or COVIDVax cohorts. A 90-day history pre-vaccination (baseline period) and ≥6 months follow-up post-vaccination were required. Event rates of new-onset hearing loss (HL), sudden HL, tinnitus, vertigo/dizziness, aural fullness, and otalgia in the 6 months post-vaccination were compared between groups after high-dimensional propensity score (hdPS) matching. A sensitivity analysis was conducted among patients with no COVID-19 infection at any time. Odds ratios (ORs) were calculated using logistic regression for the hdPS matched cohorts.

**Results:**

After hdPS matching, 20,325 patients were included into the FluVax and COVIDVax cohorts, respectively (mean age: 65.5 and 65.2 years; 53.1 and 53.8% females). The rates of otologic AEs in the 6 months post-vaccination were similarly low for the FluVax and COVIDVax cohorts: 1.16% vs. 1.16% for any HL, 0.01% vs. 0.02% for sudden HL, 0.41% vs. 0.47% for tinnitus, 1.96% vs. 1.59% for vertigo, 0.27% vs. 0.25% for otalgia, and 0.09% vs. 0.2% for aural fullness. COVIDVax patients had lower odds of vertigo [OR 95% CI: 0.81 (0.70, 0.94)] and higher odds of aural fullness [2.16 (1.25–3.72)] than the FluVax patients (both *p* < 0.05). The results of the sensitivity analysis limited to patients with no COVID-19 infection at any time (*N* = 17,530 each cohort) were consistent with the primary results, but aural fullness was the only AE with statistically higher risk in the COVIDVax vs. FluVax cohort [OR (95% CI): 1.90 (1.09–3.31); *p* = 0.021].

**Conclusion:**

New-onset otologic AEs were rare among a large cohort of hdPS-matched patients who received mRNA COVID-19 or pre-pandemic flu vaccination at a single institution. Although aural fullness was statistically more common in the COVIDVax vs. FluVax cohort, regardless of COVID-19 infection status, it remained extremely rare (<0.22%) in any cohort. These results indicate a similar otologic safety profile of the two vaccines, although future research is recommended in larger EHR databases to corroborate the findings.

## 1 Introduction

The COVID-19 pandemic began in December 2019 and by January 11, 2020, the genetic sequence of severe acute respiratory syndrome coronavirus 2 (SARS-CoV-2) was identified (1, 2). Within less than a year, in December 2020, the United States (US) Food Drug Administration (FDA) issued the first Emergency Use Authorizations for two bivalent mRNA SARS-CoV-2 vaccines from Pfizer-BioNTech (BNT162b2) and Moderna (mRNA-1273) (3), based on robust efficacy and safety evidence from large clinical trials (4, 5). Although additional vaccines have since become available (6, 7), as of August 2024, the Pfizer-BioNTech or Moderna mRNA vaccines have accounted for the large majority (>97%) of COVID-19 vaccinations in the US (8). These vaccines are highly effective in preventing moderate to severe COVID-19 symptoms and reducing related hospitalizations and mortality (9, 10), saving an estimated 1.6 million lives during 2020–2023 (11).

Otologic adverse events (AEs), such as sensorineural hearing loss (SNHL), tinnitus, vertigo, otalgia (ear pain), and aural fullness, were not identified as potential AEs of the bivalent mRNA COVID-19 vaccines in their clinical trials (12, 13). However, as the pandemic advanced, case reports began to emerge regarding possible otologic AEs following SARS-CoV-2 infection (14–18) as well as COVID-19 vaccination (19–25). In parallel, otologic AEs post-COVID-19 vaccination appeared in pharmacovigilance databases at higher-than-expected frequencies, although it was unclear whether this was related to the novelty of the pandemic, coincidence, or an actual elevation. The World Health Organization (WHO) conducted screening of otologic AEs occurring after COVID-19 vaccination in VigiBase and noted elevated reporting of hearing loss and tinnitus in February and November 2021 (26). Subsequently, a query of the FDA’s Vaccine Adverse Event Reporting System database for otologic AEs after COVID-19 vaccination found that signal ratios reached significance levels for tinnitus and vertigo, but not hearing loss, and that few events were clinically significant (27).

The results of retrospective cohort studies examining the rates of otologic AEs after COVID-19 vaccination have also been mixed, with smaller, earlier studies observing elevated incidence that was not consistently observed when using large national databases or after adjusting for confounders. A 2021 chart review by the House Ear Clinic identified elevated incidence of new-onset sudden SNHL diagnoses during the same 30-day period in 2019 and 2021 (1.6% vs. 3.8%), but the number of COVID-19 vaccinated patients presenting with otologic AEs was low (28). Specifically, just 30 of 1,325 clinical visits in 2021 had new or worsened otologic symptoms that began shortly after mRNA COVID-19 vaccination, and 11 of these patients had pre-existing otologic conditions like Meniere’s and/or autoimmune inner ear disease (28). An Israeli cohort study of 2.6 million patients observed a small increase in the risk of sudden SNHL occurring within 21 days after the first or second Pfizer-BioNTech mRNA vaccine dose as compared to the expected case rate in 2018, but not in 2019 (29). An Australian cohort study of around 5 million records noted higher incidence of tinnitus and vertigo in the 42 days after vaccination relative to all other pre- and post-vaccination time windows (30). Conversely, a Finnish cohort study of 5.5 million people, which adjusted for potential confounders like comorbidities, observed no increased risk of sudden SNHL within 54 days after COVID-19 vaccination versus pre-pandemic levels (31). Similarly, a Malaysian cohort study of age-matched adults with and without sudden SNHL during January 25, 2020 and June 30, 2022 reported similar rates of COVID-19 vaccination across the groups (32).

Although causality has not yet been definitively established in the hypervigilant setting of a new pandemic, the medical and scientific communities recognized the importance of delineating the otologic safety profile of the novel COVID-19 vaccines. Electronic health records (EHR) provide a rich data source for assessing the frequency of new-onset otologic AEs following vaccination, enabling comparisons of the rates associated with vaccination for COVID-19 versus other vaccines with high uptake. To this end, we assessed and compared the rates of emergent hearing loss, tinnitus, vertigo, otalgia, and aural fullness in adults who received an mRNA COVID-19 vaccine versus similar adults receiving any influenza vaccine prior to the pandemic. To ensure a fair comparison, patients were matched 1:1 on demographics and comorbidities using high-dimensional propensity score (hdPS) methods. To examine whether COVID-19 infection impacted the risk of otologic AEs post-COVID-19 vaccination, we also conducted a sensitivity analysis restricted to patients with no history of COVID-19 infection at any time.

## 2 Methods and materials

### 2.1 Data source

This retrospective cohort study used limited EHR data collected during routine care at Stanford Health Care (SHC) in Palo Alto, CA, US during 2016 to 2022. The EHR contained the demographics, diagnostic codes, medications, procedures, and limited dates of medical services for approximately 1,070,00 patients during the study period. Given that the source dataset was de-identified, without the ability to link to the source individuals, this analysis was considered “non-human subject” research and no ethical review was required per institutional policy.

### 2.2 Participants and cohorts

Patients in the EHR were eligible for the study if they received a Pfizer/Moderna COVID-19 vaccination (December 2020 to January 2022) or any influenza vaccine (pre-pandemic years of January 2016 to December 2019), were aged 50 to 89 years on the date of vaccination (index date), and had no diagnosis of otologic disorders prior to the index date (see Supplementary Table S1 for the list of diagnostic codes). A 90-day history pre-vaccination (baseline period) and ≥6 months of follow-up post-vaccination (study period) were required for inclusion. For patients receiving an mRNA COVID-19 vaccine, their index date was the date of the first vaccine. Patients younger than 50 years were excluded in order to maximize the success of the matching on comorbidities, given that older people were prioritized for COVID-19 vaccination early in the pandemic, and to reduce the risk of including patients with genetic hearing loss appearing in childhood or young adulthood.

Patients were categorized into two non-overlapping cohorts based on if they received a mRNA COVID-19 vaccine (COVIDVax cohort) or any flu vaccine (FluVax cohort). COVID-19 vaccination was identified with Current Procedural Terminology (CPT) code 90471. The list of flu vaccine serotypes with identifier codes is in Supplementary Table S2.

### 2.3 Outcomes

The following otologic AEs were identified by International Classification of Diseases 9th (ICD9) or 10th edition (ICD10) diagnostic codes during the 6-month period post-vaccination: hearing loss (ICD9: 389 and ICD10: H90, H91); sudden hearing loss (ICD9: 388.2, ICD10: H91.2), tinnitus (ICD9: 386, 780.4 and ICD10: H81, R42); otalgia (ICD9: 388.7, ICD10: H92.0); vertigo (ICD9: 386, 780.4 and ICD10: H81, R42); and aural fullness (ICD9: 388.8, ICD10: H93.8, H93.9). Vertigo included diagnostic terms for both dizziness and vertigo per the consensus that distinguishing between the terms is of limited clinical utility (33).

### 2.4 Propensity score matching

Potential baseline confounders were identified using diagnostic codes observed in the 90 days prior to cohort entry, including demographic characteristics and comorbidities included in the Charlson Comorbidity Index (CCI) (34, 35). Patients in the COVIDVax cohort were matched 1:1 with patients in the FluVax cohort via hdPS matching on age, sex, CCI score, diagnostic codes, procedure codes, medication codes, and number of and type of health system encounters, as previously described by Schneeweiss et al. (36). These variables were used as covariates in a logistic regression model to estimate propensity scores. Greedy matching with 0.25 caliper width was used. The standard deviation (SD) of logit propensity score was used for obtaining 1:1 matching of the two groups. Absolute standardized differences were estimated to assess balance after matching and variables, with standard mean differences (SMD) < 0.1 between the cohorts determined to be good matches.

### 2.5 Statistical analysis

The event rates of each otologic AE during the 6-month period post-vaccination were calculated by cohort. Odds ratios (ORs) with 95% confidence intervals (CIs) were calculated with logistic regression in the hdPS-matched cohorts to report the magnitude of the difference in rates of otologic AEs. The primary analysis was repeated in a sensitivity analysis among patients who also had no history of COVID-19 infection (ICD10: U07.1) at any time.

All analyses were performed using R (v4.2.1, R Core Team 2022) and the gtsummary package (v 1.7.2) on the Atropos Health platform. A *p* < 0.01 was used to determine statistical significance.

## 3 Results

### 3.1 Cohort selection and baseline characteristics

#### 3.1.1 Primary cohort

A total of 42,859 patients who received influenza vaccination during 2016–2019 and 30,434 non-overlapping patients who received mRNA COVID-19 vaccination during 2020–2022, with no prior history of otologic disorders and aged 50 to 89 years, were identified in the SHC database. After hdPS matching, 20,325 patients were included into each cohort.

Prior to matching, the FluVax and COVIDVax cohorts differed on multiple variables (i.e., SMD ≥ 0.01), including mean age, age distribution, race distribution, proportion with Hispanic ethnicity, duration of history in the EHR, and proportions with malignancy and peripheral vascular disease (Table 1). After matching, the characteristics of the FluVax and COVIDVax cohorts were generally balanced (all matched-on items SMD < 0.01), with a mean age of 65.5 vs. 65.2 years, respectively, 53.1% vs. 53.8% females, 61.9% vs. 57.3% White, similar CCI scores (3.5 vs. 3.4), and similar rates of all CCI comorbidities (Table 1). Differences that persisted between the cohorts post-matching included the proportions of patients aged 70–79 years [19.6% (FluVax) vs. 25.3% (COVIDVax)] and 80–89 years (9.1% vs. 5.2%), the proportions who were Asian (17.5% vs. 22.9%) or Black (5.2% vs. 3.1%), and the mean pre-index days (3,121.5 vs. 3,988.8) and follow-up days (1,304.3 vs. 374.8) in the EHR.

**Table 1:** Baseline demographic and clinical characteristics of the COVID-19 and flu vaccine cohorts (primary analysis), before and after hdPS matching.

	Before matching			After matching		
	FLUVAX *N* = 42,859	COVIDVAX *N* = 30,434	SMD	FLUVAX *N* = 20,325	COVIDVAX *N* = 20,325	SMD
Demographics						
Female, *n* (%)	23,143 (54%)	16,437 (54%)	0.0002	10,787 (53.1%)	10,931 (53.8%)	0.0142
Mean age, years (SD)	62 (9.0)	67.9 (9.2)	0.6554	65.5 (9.4)	65.2 (8.9)	0.0240
Age range, *n* (%)						
50–59 years	20,850 (48.6%)	6,714 (22.10%)	0.5701	6,175 (30.4%)	6,272 (30.9%)	0.0104
60–69 years	14,238 (33.2%)	10,585 (34.80%)	0.0330	8,321 (40.9%)	7,863 (38.7%)	0.0460
70–79 years	5,537 (12.9%)	10,226 (33.60%)	0.5196	3,981 (19.6%)	5,133 (25.3%)	0.1362
80–89 years	2,234 (5.2%)	2,909 (9.60%)	0.1707	1848 (9.1%)	1,057 (5.2%)	0.1515
Race, *n* (%)						
White	25,701 (60%)	18,011 (59.2%)	0.0160	12,575 (61.9%)	11,654 (57.3%)	0.0924
Asian	7,736 (18%)	6,792 (22.3%)	0.1072	3,566 (17.5%)	4,652 (22.9%)	0.1333
Other	7,062 (16.5%)	4,676 (15.4%)	0.0303	3,118 (15.3%)	3,384 (16.6%)	0.0357
Black	2,360 (5.5%)	955 (3.1%)	0.1141	1,066 (5.2%)	635 (3.1%)	0.1061
Hispanic ethnicity, *n* (%)	3,775 (8.8%)	1,845 (6.1%)	0.1033	1,621 (8%)	1,312 (6.5%)	0.0588
History in EHR						
Mean pre-index days (SD)	3075.7 (2164.6)	4051.2 (2618.8)	0.4127	3121.5 (2219.8)	3988.8 (2623.1)	0.3569
Mean follow-up days (SD)	1341.4 (563.7)	382.7 (216.4)	2.1161	1304.3 (570.6)	374.8 (218.1)	2.1519
Mean encounters (SD)	19.8 (34.6)	8.4 (21.3)	0.3815	9.1 (23.5)	9.6 (23.7)	0.0184
Comorbidities						
Mean CCI score (SD)	3.1 (2.6)	3.9 (2.8)	0.2985	3.5 (2.7)	3.4 (2.6)	0.0199
CCI comorbidities, *n* (%)						
Malignancy	4,938 (11.52%)	5,283 (17.36%)	0.1691	2,545 (12.52%)	3,047 (14.99%)	0.0718
Metastatic solid tumor	1,146 (2.67%)	1,198 (3.94%)	0.0718	555 (2.73%)	673 (3.31%)	0.0339
Diabetes	7,433 (17.34%)	4,405 (14.47%)	0.0780	3,400 (16.73%)	2,689 (13.23%)	0.0981
Diabetes with complications	2,722 (6.35%)	1790 (5.88%)	0.0195	1,304 (6.42%)	1,038 (5.11%)	0.0562
Congestive heart failure	2037 (4.75%)	2086 (6.85%)	0.0913	1,080 (5.31%)	1,093 (5.38%)	0.0028
Myocardial infarction	1,060 (2.47%)	1,146 (3.77%)	0.0757	530 (2.61%)	639 (3.14%)	0.0321
Peripheral vascular disease	1848 (4.31%)	2,130 (7%)	0.1188	1,023 (5.03%)	1,167 (5.74%)	0.0314
Chronic pulmonary disease	6,990 (16.31%)	4,080 (13.41%)	0.0811	3,118 (15.34%)	2,502 (12.31%)	0.0879
Cerebrovascular disease	2,174 (5.07%)	2,163 (7.11%)	0.0863	1,223 (6.02%)	1,137 (5.59%)	0.0181
Dementia	463 (1.08%)	383 (1.26%)	0.0167	341 (1.68%)	161 (0.79%)	0.0803
Hemiparaplegia	333 (0.78%)	247 (0.81%)	0.0039	156 (0.77%)	144 (0.71%)	0.0069
Mild liver disease	3,039 (7.09%)	2,589 (8.51%)	0.0532	1,325 (6.52%)	1,617 (7.96%)	0.0555
Severe liver disease	318 (0.74%)	214 (0.70%)	0.0046	130 (0.64%)	109 (0.54%)	0.0135
Renal disease	3,136 (7.32%)	2,812 (9.24%)	0.0705	1715 (8.44%)	1,538 (7.57%)	0.0321
Peptic ulcer disease	584 (1.36%)	539 (1.77%)	0.0332	269 (1.32%)	311 (1.53%)	0.0174
Rheumatic disease	1,305 (3.04%)	987 (3.24%)	0.0114	625 (3.08%)	610 (3%)	0.0043
HIV	157 (0.37%)	49 (0.16%)	0.0388	68 (0.33%)	41 (0.20%)	0.0257

Blue indicates SMD ≥0.1 and that the characteristic is unbalanced between the cohorts. Abbreviations: CCI, Charlson Comorbidity Index; EHR, electronic health records; HIV, human immunodeficiency virus; hdPS, high-dimensional propensity score; SD, standard deviation; SMD, standardized mean difference.

#### 3.1.2 Sensitivity analysis cohort: patients with no COVID-19 infection at any time

The primary analysis was repeated among patients meeting all study criteria but restricting the COVIDVax cohort to patients with no diagnosis of COVID-19 infection at any time. A total of 40,390 and 25,556 eligible patients with flu and COVID-19 vaccination, respectively, were identified. After hdPS matching, 17,530 patients were included into the FluVax and COVIDVax cohorts. Most characteristics were balanced between the cohorts after matching, including the mean age [66.3 (FluVax) vs. 66.0 years (COVIDVax)], proportion of females (53.9% vs. 54.4%), CCI score (mean: 3.6 vs. 3.5), and the proportions with all CCI comorbidities (Supplementary Table S3). Differences that persisted post-matching were similar to those in the primary cohort and included the proportions of patients aged 70–79 and 80–89 years, the proportions who were Asian or Black, and the mean number of pre-index and follow-up days. As the mean age and CCI, and the sex and racial distributions, were similar to those in the primary cohort, we used this cohort as sensitivity analysis among COVID-19 infection-naïve patients.

### 3.2 Rates of otologic AEs during the 6 months following vaccination

#### 3.2.1 Primary analysis

The event rates and odds of emergent otologic AEs in the 6 months following flu or COVID-19 vaccination in the hdPS-matched cohorts are listed in Table 2. Post-vaccination otologic AEs were rare in both cohorts: 1.16% vs. 1.16% for any hearing loss in the FluVax and COVIDVax cohorts, respectively, 0.01% vs. 0.02% for sudden hearing loss, 0.41% vs. 0.47% for tinnitus, 1.96% vs. 1.59% for vertigo, 0.27% vs. 0.25% for otalgia, and 0.09% vs. 0.20% for aural fullness. There were no significant differences between the FluVax and COVIDVax cohorts in the odds of any hearing loss [OR (95% CI): 1.0 (0.83, 1.2)], sudden hearing loss [2.50 (0.41, 26.3)], tinnitus [1.13 (0.84, 1.52)], or otalgia [0.93 (0.63, 1.36)] (all *p* > 0.05). The odds of vertigo were statistically lower in the COVIDVax compared to the FluVax cohort [OR (95% CI): 0.81 (0.70, 0.94), *p* = 0.005], while the odds of aural fullness were significantly higher [2.16 (1.25, 3.72), *p* = 0.004].

**Table 2:** Rates of emergent otologic AEs in the 6 months following flu or COVID-19 vaccination, after hdPS matching.

Primary analysis	FluVax *N* = 20,325	COVIDVax *N* = 20,325	OR (95% CI)	*P*
Any hearing loss	236 (1.16%)	236 (1.16%)	1.00 (0.83, 1.2)	1.000
Sudden hearing loss	2 (0.01%)	5 (0.02%)	2.50 (0.41, 26.3)	0.453
Tinnitus	84 (0.41%)	95 (0.47%)	1.13 (0.84, 1.52)	0.410
Vertigo	399 (1.96%)	324 (1.59%)	0.81 (0.70, 0.94)	0.005*
Otalgia	55 (0.27%)	51 (0.25%)	0.93 (0.63, 1.36)	0.697
Aural fullness	19 (0.09%)	41 (0.2%)	2.16 (1.25, 3.72)	0.004*

Sensitivity analysis (no COVID-19 infection)	*N* = 17,530	*N* = 17,530		
Any hearing loss	202 (1.15%)	223 (1.27%)	1.11 (0.91, 1.34)	0.305
Sudden hearing loss	2 (0.01%)	6 (0.03%)	3.00 (0.54, 30.4)	0.289
Tinnitus	71 (0.41%)	89 (0.51%)	1.26 (0.92, 1.72)	0.153
Vertigo	342 (1.95%)	306 (1.75%)	0.89 (0.76, 1.04)	0.153
Otalgia	46 (0.26%)	50 (0.29%)	1.09 (0.73, 1.62)	0.683
Aural fullness	19 (0.11%)	36 (0.21%)	1.90 (1.09, 3.31)	0.021*

AE, adverse event; CI, confidence interval; hdPS, high-dimensional propensity score; OR, odds ratio. *p < 0.05.

#### 3.2.2 Sensitivity analysis: patients with no COVID-19 infection at any time

After hdPS matching (*N* = 17,530 each cohort), the rates of otologic AEs post-vaccination among patients with flu vaccination versus with COVID-19 vaccination and no COVID-19 infection were consistent with those observed in the primary analysis: 1.15% vs. 1.27% for any hearing loss, respectively, 0.01% vs. 0.03% for sudden hearing loss, 0.41% vs. 0.51% for tinnitus, 1.95% vs. 1.75% for vertigo, 0.26% vs. 0.29% for otalgia, and 0.11% vs. 0.21% for aural fullness (Table 2). The only significant difference in the odds of otologic AEs was for aural fullness, which was significantly higher in the COVIDVax cohort compared with the FluVax cohort [OR 95% CI: 1.90 (1.09, 3.31), *p* = 0.021].

## 4 Discussion

The results of this single-institution, retrospective cohort study among adults aged 50 to 89 years suggests that the mRNA COVID-19 vaccines have a similar otologic safety profile to that of flu vaccines. The rates of any emergent otologic AEs in the 6 months after vaccination were low (<2.0%) with either vaccine, with the most common AE being vertigo in both the COVIDVax (1.59%) and FluVax (1.96%) cohorts. More serious otologic AEs such as hearing loss, sudden hearing loss, and tinnitus were rarer and the rates did not significantly differ by vaccination cohort. In the primary analysis, the odds of vertigo were significantly lower in the COVIDVax vs. FluVax cohort, while the odds of aural fullness were higher. The results of the sensitivity analysis conducted among COVID-19 vaccinated patients with no COVID-19-positive tests were consistent with the primary results, although the odds of vertigo no longer significantly differed between the cohorts.

Aural fullness, or the sensation of pressure in the ear (37), was the only otologic AE that statistically occurred with more frequency in COVIDVax cohort compared with the FluVax cohort, regardless of COVID-19 infection status. Aural fullness is a subjective symptom that is common in patients with Eustachian tube dysfunction, commonly due to upper respiratory tract infections, allergic rhinitis, adenoid hypertrophy, among others (37, 38). It has also been observed in patients with underlying Meniere’s disease, migraine, and temporomandibular joint disorders (39–43). While it is possible that the SARS-CoV-2 virus could infect the mucosa of the Eustachian tube to cause inflammation and dysfunction (44, 45), a significantly higher rate of aural fullness was also observed among patients with no positive COVID-19 tests in this study. Interestingly, Eustachian tube dysfunction has been reported among healthcare providers (HCPs) due to quarantine masking during the COVID-19 pandemic (46–48). HCPs were the first group to be prioritized for COVID-19 vaccination and their uptake remained high (~90%) throughout the pandemic, even exceeding their flu vaccination rates (76–80%) during 2020–2022 (49). These rates are higher than those of the general US population; therefore, it is possible that HCPs engaged in enhanced masking may be enriched in the COVID-19 vaccine groups of this study and contribute to the difference in the rate of aural fullness between groups. Importantly, despite this statistical elevation in the rate of aural fullness, the proportions of patients who experienced this AE were very low (<0.22%) in any cohort, occurring in just 19 of 20,325 patients in the FluVax cohort and 41 of 20,325 in the COVIDVax cohort.

Sudden hearing loss was the rarest otologic AE observed in this study, occurring in only 0.01% (*n* = 2/20,325) and 0.02% (*n* = 5/20,325) and 0.02% of the COVIDVax and FluVax cohorts, respectively, and did not significantly differ between cohorts in the primary or sensitivity analysis. This finding is notable given the early attention attracted by case reports and some cohort studies reporting elevated rates of the AE following COVID-19 vaccination (28, 29), but is consistent with the results of studies in Finland and Malaysia that observed no increased risk (31, 32). Further, the current findings are consistent with a 2023 meta-analysis of 11 observational studies that reported similar incidence of post-vaccination SNHL for COVID-19 and non-COVID-19 vaccines (50). Nevertheless, the wide CIs in the present comparisons on this outcome indicate a level of uncertainty, requiring future studies in larger datasets.

The rates of new-onset tinnitus were similarly low among patients receiving either the flu or mRNA COVID-19 vaccine (0.41–0.51% across cohorts), which is consistent with a recent study examining reports of tinnitus after COVID-19 vaccination in VAERS during 2020–2024 (51). That study did not find disproportionate reporting of tinnitus after COVID-19 vaccination in VAERS, and reported that the incidence rates were similar to those after flu vaccination except for people aged ≥65 years (51). Therefore, age may be a potential risk factor for otologic AEs following COVID-19 vaccination, although the relationship requires further research as older age is also an independent risk factor for hearing loss and tinnitus (52–54). Additionally, a recent study has suggested that people with metabolic disorders may be at higher risk of tinnitus post-COVID-19 vaccination (55), therefore future research is needed to examine the rates of otologic events occurring in high-risk populations with certain comorbidities. Of note, our study did not assess worsening of pre-existing tinnitus, and a 2025 patient-reported survey study of 372 people with new-onset or pre-existing tinnitus following COVID-19 vaccination found that approximately half (53%) of those with pre-existing tinnitus experienced worsening of symptoms after vaccination (56). However, pandemic-related anxiety was identified a contributing factor to the exacerbation of the respondents’ tinnitus in that study (56), a relationship which has been previously noted for anxiety in general (57).

A notable strength of this analysis is the use of hdPS matching which controlled for potential confounders in the baseline demographic and clinical characteristics of the cohorts. This included controlling for cerebrovascular disease, malignancy, cardiovascular disorders (i.e., myocardial infarction, congestive heart failure, and peripheral vascular disease), and mild liver disease, which are all risk factors for hearing loss and could otherwise bias the comparison (58–61). Matching the cohorts on age is also essential given that the risk of hearing loss and tinnitus increases with age (53, 62–65). We also matched on CCI score because a higher score increases the likelihood of poor outcomes following COVID-19 infection (66), although the relationship with otologic AE risk has not yet been established.

This study is subject to several limitations, some of which are inherent to retrospective studies using EHR data such as the potential for mis-coding and the presence of rule-out diagnosis codes. First, this study only captured diagnoses, vaccine administration events, and other medical services that were provided at SHC. Healthcare services, including vaccine administrations and COVID-19 tests, provided at other facilities would not be captured. This caveat is notable given the large number of people who received vaccination or testing at government clinics or quick-care pharmacies during the COVID-19 pandemic (67). We mitigated this by limiting our analysis to patients receiving their first COVID-19 vaccination, although it is possible that patients may have received a second or booster dose during the 6-month follow-up period. Similarly, the sensitivity analysis restricted to patients with no positive COVID-19 tests reflects testing performed at SHC.

Second, as the analyses were conducted in people aged 50 to 89 years receiving care at a single institution in Palo Alto, California, the results may not generalize to younger people or areas of the US with differing demographics. For example, the racial composition of the main hdPS-matched cohorts was 18–23% Asian and 7–8% Black, while the general US population is approximately 6% Asian and 19% Black per the 2020 Census (68). Third, the cohorts were non-overlapping by design to prevent double-counting of patients. However, this also means that the FluVax cohort was not subject to pandemic-specific factors, such as potential psychosocial impacts on tinnitus (69) and general alterations in health-seeking behavior and access (70, 71). Fourth, a general limitation of pharmacovigilance studies is the risk of masking bias, in which the novelty of a treatment and unprecedented rates of reporting can lead to overestimation of the true rate of AEs (72). Similarly, because patients may be more likely to receive low-impact health services like vaccination or COVID-19 tests at quick-care clinics or pharmacies, but more likely to receive diagnostic services for otologic events at hospitals and academic clinics like SHC, it is possible that the rates of otologic events may be over-estimated in the study populations as only services at SHC were captured. Fifth, the EHR database did not contain information on patients’ genetics or family history related to otologic events. Future studies using databases containing such information would be valuable for validating the results. Finally, this analysis only examined mRNA COVID-19 vaccines due their high uptake in the US; the rates of otologic AEs with other COVID-19 vaccines were not assessed.

In conclusion, the results of this retrospective analysis of EHR data indicate that mRNA COVID-19 vaccines and flu vaccines have similar otologic safety profiles, although may be associated with AEs in a small proportion of these vaccinated populations. The rates of otologic AEs were very low in all cohorts assessed and only aural fullness was statistically more common among patients receiving mRNA COVID-19 versus flu vaccination in both the primary and sensitivity analyses. Although these findings are reassuring, particularly regarding the similarly low rates of severe events like hearing loss and tinnitus, future research using larger, multi-institutional EHR databases is recommended to corroborate these results and continue to delineate the full safety profile of these globally important vaccines.

## Data Availability

The original contributions presented in the study are included in the article/Supplementary material, further inquiries can be directed to the corresponding author.

## Supplementary material

SUPPLEMENTARY TABLE S1 Otologic diagnoses used as exclusion criteria for the COVID-19 vaccination and influenza vaccination cohorts.

SUPPLEMENTARY TABLE S2 Influenza vaccine serotypes identified by Concept Unique Identifier (RxCUI).

SUPPLEMENTARY TABLE S3 Baseline demographic and clinical characteristics of the COVID-19 and flu vaccine cohorts with no history of COVID-19 infection (Sensitivity Analysis), before and after hdPS matching.
