# Supplementary material for: Comparison of the rates of emergent otologic adverse events following mRNA COVID-19 versus influenza vaccination: a matched cohort analysis
Source: Front Neurol. 2025 Aug 7;16:1637870. doi: 10.3389/fneur.2025.1637870 (PMC12367506; doi:10.3389/fneur.2025.1637870)
Supplement: Supplementary file 1 [file Table_1.docx]

**SUPPLEMENTARY MATERIAL**

**Table S1. Otologic diagnoses used as exclusion criteria for the COVID-19 vaccination and influenza vaccination cohorts**

| **ICD code** | **Diagnosis** |
| --- | --- |
| **ICD9** |  |
| [380] | Disorders of external ear |
| [381] | Nonsuppurative otitis media and Eustachian tube disorders [382] Suppurative and unspecified otitis media |
| [383] | Mastoiditis and related conditions |
| [384] | Other disorders of tympanic membrane |
| [385] | Other disorders of middle ear and mastoid |
| [386] | Vertiginous syndromes and other disorders of vestibular system |
| [387] | Otosclerosis |
| [388] | Other disorders of ear |
| [389] | Hearing loss |
| [390] | Rheumatic fever without mention of heart involvement |
| **ICD10** |  |
| [H60] | Otitis externa |
| [H61] | Other disorders of external ear |
| [H62] | Disorders of external ear in diseases classified elsewhere |
| [H65] | Nonsuppurative otitis media |
| [H66] | Suppurative and unspecified otitis media |
| [H67] | Otitis media in diseases classified elsewhere |
| [H68] | Eustachian salpingitis and obstruction |
| [H69] | Other and unspecified disorders of Eustachian tube |
| [H70] | Mastoiditis and related conditions |
| [H71] | Cholesteatoma of middle ear |
| [H72] | Perforation of tympanic membrane |
| [H73] | Other disorders of tympanic membrane |
| [H74] | Other disorders of middle ear mastoid |
| [H75] | Other disorders of middle ear and mastoid in diseases classified elsewhere |
| [H80] | Otosclerosis |
| [H81] | Disorders of vestibular function |
| [H82] | Vertiginous syndromes in diseases classified elsewhere |
| [H83] | Other diseases of inner ear |
| [H90] | Conductive and sensorineural hearing loss |
| [H91] | Other and unspecified hearing loss |
| [H92] | Otalgia and effusion of ear |
| [H93] | Other disorders of ear, not elsewhere classified |
| [H94] | Other disorders of ear in diseases classified elsewhere |
| [H95] | Intraoperative and postprocedural complications and disorders of ear and mastoid process, not elsewhere classified |

**Table S2. Influenza vaccine serotypes identified by Concept Unique Identifier (RxCUI)**

| **RxCUI** | **Vaccine(s)** |
| --- | --- |
| [1005911] | Influenza Virus Vaccine, Live Attenuated, A-Perth-16-2009 (H3N2) strain |
| [1005914] | Influenza Virus Vaccine, Live Attenuated, A-California-7-2009 (H1N1) strain 158000 000 UNT/ML / Influenza Virus Vaccine, Live Attenuated, A-Perth-16-2009 (H3N2) strain 158000 000 UNT/ML / Influenza Virus Vaccine, Live Attenuated, B-Brisbane-60-2008 strain 1 |
| [1005931] | Influenza Virus Vaccine, Inactivated A-Victoria-210-2009 X-187 (H3N2) (A-Perth-16- 2009) strain |
| [1005951] | 0.5 ML Influenza Virus Vaccine, Inactivated A-California-07-2009 X-179A (H1N1) strain 0.12 MG/ML / Influenza Virus Vaccine, Inactivated A-Victoria-210-2009 X-187 (H3N2) (A-Pert h-16-2009) strain 0.12 MG/ML / Influenza Virus Vaccine, Inactivated B-Brisbane- |
| [1111370] | 0.1 ML Influenza Virus Vaccine, Inactivated A-California-07-2009 X-179A (H1N1) stra in 0.09 MG/ML / Influenza Virus Vaccine, Inactivated A-Victoria-210-2009 X-187 (H3N2) (A-Pert h-16-2009) strain 0.09 MG/ML / Influenza Virus Vaccine, Inactivated B-Brisbane- |
| [1116742] | 0.5 ML Influenza Virus Vaccine, Inactivated A-Christchurch-16-2010 NIB-74 (H1N1) ( A-California-7-2009) strain 0.03 MG/ML / Influenza Virus Vaccine, Inactivated A-Victoria-210-2 009 X-187 (H3N2) (A-Perth-16-2009) strain 0.03 MG/ML / Influenza Virus Vaccine, |
| [1116750] | Influenza Virus Vaccine, Inactivated A-Christchurch-16-2010 NIB-74 (H1N1) (A-Calif ornia-7-2009) strain 0.03 MG/ML / Influenza Virus Vaccine, Inactivated A-Victoria-210-2009 X-1 87 (H3N2) (A-Perth-16-2009) strain 0.03 MG/ML / Influenza Virus Vaccine, Inactive |
| [1303855] | Influenza B virus vaccine, B-Wisconsin-1-2010-like virus |
| [1304122] | Influenza A virus vaccine, A-Victoria-361-2011 (H3N2)-like virus |
| [1304128] | 0.5 ML Influenza A virus vaccine, A-California-7-2009 (H1N1)-like virus 0.03 MG/ML / Influenza A virus vaccine, A-Victoria-361-2011 (H3N2)-like virus 0.03 MG/ML / Influenza B virus vaccine, B-Wisconsin-1-2010-like virus 0.03 MG/ML Prefilled Syringe |
| [1304182] | Influenza A virus vaccine, A-California-7-2009 (H1N1)-like virus 0.03 MG/ML / Influenza A virus vaccine, A-Victoria-361-2011 (H3N2)-like virus 0.03 MG/ML / Influenza B virus vaccine, B-Wisconsin-1-2010-like virus 0.03 MG/ML Injectable Suspension |
| [1304261] | Influenza A virus vaccine, A-California-7-2009 (H1N1)-like virus 158000000 UNT/ML / Influenza A virus vaccine, A-Victoria-361-2011 (H3N2)-like virus 158000000 UNT/ML / Influenza B virus vaccine, B-Wisconsin-1-2010-like virus 158000000 UNT/ML Nasal Spray |
| [1427022] | Influenza B virus vaccine, B-Massachusetts-2-2012-like virus |
| [1427027] | Influenza A virus vaccine, A-California-7-2009 (H1N1)-like virus 0.03 MG/ML / Influenza A virus vaccine, A-Victoria-361-2011 (H3N2)-like virus 0.03 MG/ML / Influenza B virus vaccine, B-Brisbane-60-2008-like virus 0.03 MG/ML / Influenza B virus vaccine, B- |
| [1427051] | 0.25 ML Influenza A virus vaccine, A-California-7-2009 (H1N1)-like virus 0.03 MG/M L / Influenza A virus vaccine, A-Victoria-361-2011 (H3N2)-like virus 0.03 MG/ML / Influenza B vi rus vaccine, B-Brisbane-60-2008-like virus 0.03 MG/ML / Influenza B virus vac |
| [1428988] | 0.5 ML Influenza A virus vaccine, A-California-7-2009 (H1N1)-like virus 0.03 MG/ML / Influenza A virus vaccine, A-Victoria-361-2011 (H3N2)-like virus 0.03 MG/ML / Influenza B virus vaccine, B-Massachusetts-2-2012-like virus 0.03 MG/ML Prefilled Syringe |
| [1429570] | Influenza A virus vaccine, A-California-7-2009 (H1N1)-like virus 0.03 MG/ML / Influenza A virus vaccine, A-Victoria-361-2011 (H3N2)-like virus 0.03 MG/ML / Influenza B virus vaccine, B-Massachusetts-2-2012-like virus 0.03 MG/ML Injectable Suspension |
| [1429577] | 0.1 ML Influenza A virus vaccine, A-California-7-2009 (H1N1)-like virus 0.09 MG/ML / Influenza A virus vaccine, A-Victoria-361-2011 (H3N2)-like virus 0.09 MG/ML / Influenza B virus vaccine, B-Massachusetts-2-2012-like virus 0.09 MG/ML Prefilled Syringe |
| [1429590] | 0.5 ML Influenza A virus vaccine, A-California-7-2009 (H1N1)-like virus 0.12 MG/ML / Influenza A virus vaccine, A-Victoria-361-2011 (H3N2)-like virus 0.12 MG/ML / Influenza B viru s vaccine, B-Massachusetts-2-2012-like virus 0.12 MG/ML Prefilled Syringe |
| [1429603] | 0.25 ML Influenza A virus vaccine, A-California-7-2009 (H1N1)-like virus 0.03 MG/M L / Influenza A virus vaccine, A-Victoria-361-2011 (H3N2)-like virus 0.03 MG/ML / Influenza B virus vaccine, B-Massachusetts-2-2012-like virus 0.03 MG/ML Prefilled Syringe |
| [1442205] | Influenza A virus vaccine, A-California-7-2009 (H1N1)-like virus 0.09 MG/ML / Influenza A virus vaccine, A-Victoria-361-2011 (H3N2)-like virus 0.09 MG/ML / Influenza B virus vaccine, B-Massachusetts-2-2012-like virus 0.09 MG/ML Injectable Solution |
| [1541617] | Influenza A virus vaccine, A-Texas-50-2012 (H3N2)-like virus |
| [1541622] | 0.5 ML Influenza A virus vaccine, A-California-7-2009 (H1N1)-like virus 0.03 MG/ML / Influenza A virus vaccine, A-Texas-50-2012 (H3N2)-like virus 0.03 MG/ML / Influenza B virus vaccine, B-Brisbane-60-2008-like virus 0.03 MG/ML / Influenza B virus vaccine, |
| [1543766] | Influenza A virus vaccine, A-California-7-2009 (H1N1)-like virus 50000000 MG/ML / Influenza A virus vaccine, A-Victoria-361-2011 (H3N2)-like virus 50000000 MG/ML / Influenza B virus vaccine, B-Massachusetts-2-2012-like virus 50000000 MG/ML / Influenza Vir |
| [1657128] | influenza A virus (H1N1) antigen |
| [1657131] | influenza A virus (H3N2) antigen |
| [1657134] | influenza B virus antigen |
| [1657334] | influenza A virus A/California/7/2009 (H1N1) antigen 0.03 MG/ML / influenza A virus A/Switzerland/9715293/2013 (H3N2) antigen 0.03 MG/ML / influenza B virus B/Brisbane/60/20 08 antigen 0.03 MG/ML / influenza B virus B/Phuket/3073/2013 antigen 0.03 MG/ML In |
| [1657844] | 0.5 ML influenza A virus A/California/7/2009 (H1N1) antigen 0.03 MG/ML / influenza A virus A/Switzerland/9715293/2013 (H3N2) antigen 0.03 MG/ML / influenza B virus B/Brisban e/60/2008 antigen 0.03 MG/ML / influenza B virus B/Phuket/3073/2013 antigen 0.03 M |
| [1658701] | influenza A virus A/California/7/2009 (H1N1) antigen 0.03 MG/ML / influenza A virus A/Switzerland/9715293/2013 (H3N2) antigen 0.03 MG/ML / influenza B virus B/Phuket/3073/2013 antigen 0.03 MG/ML Injectable Suspension |
| [1658711] | 0.5 ML influenza A virus A/California/7/2009 (H1N1) antigen 0.12 MG/ML / influenza A virus A/Switzerland/9715293/2013 (H3N2) antigen 0.12 MG/ML / influenza B virus B/Phuket/ 3073/2013 antigen 0.12 MG/ML Prefilled Syringe |
| [1659746] | 0.5 ML influenza A virus A/California/7/2009 (H1N1) antigen 0.03 MG/ML / influenza A virus A/Switzerland/9715293/2013 (H3N2) antigen 0.03 MG/ML / influenza B virus B/Brisban e/60/2008 antigen 0.03 MG/ML / influenza B virus B/Phuket/3073/2013 antigen 0.03 M [1659754] 0.25 ML influenza A virus A/California/7/2009 (H1N1) antigen 0.03 MG/ML / influenza A virus A/Switzerland/9715293/2013 (H3N2) antigen 0.03 MG/ML / influenza B virus B/Brisbane/60/2008 antigen 0.03 MG/ML / influenza B virus B/Phuket/3073/2013 antigen 0.03 [1659981] 0.5 ML influenza A virus A/Christchurch/16/2010 (H1N1) antigen 0.03 MG/ML / influenza A virus A/Switzerland/9715293/2013 (H3N2) antigen 0.03 MG/ML / influenza B virus B/Phuket/3073/2013 antigen 0.03 MG/ML Prefilled Syringe |
| [1660924] | 0.5 ML influenza A virus A/California/7/2009 (H1N1) antigen 0.09 MG/ML / influenza A virus A/Switzerland/9715293/2013 (H3N2) antigen 0.09 MG/ML / influenza B virus B/Phuket/ 3073/2013 antigen 0.09 MG/ML Injection |
| [1661111] | 0.1 ML influenza A virus A/California/7/2009 (H1N1) antigen 0.09 MG/ML / influenza A virus A/Switzerland/9715293/2013 (H3N2) antigen 0.09 MG/ML / influenza B virus B/Brisban e/60/2008 antigen 0.09 MG/ML / influenza B virus B/Phuket/3073/2013 antigen 0.09 M [1661802] 0.5 ML influenza A virus A/California/7/2009 (H1N1) antigen 0.03 MG/ML / influenza A virus A/South Australia/55/2014 (H3N2) antigen 0.03 MG/ML / influenza B virus B/Phuket/30 73/2013 antigen 0.03 MG/ML Prefilled Syringe |
| [1664475] | influenza A virus A/Bolivia/559/2013 (H1N1) antigen 50000000 MG/ML / influenza A virus A/Switzerland/9715293/2013 (H3N2) antigen 50000000 MG/ML / influenza B virus B/Bris bane/60/2008 antigen 50000000 MG/ML / influenza B virus B/Phuket/3073/2013 antigen 50 [1724010] 0.5 ML influenza A virus A/California/7/2009 (H1N1) antigen 0.015 MG/ML / influenza A virus A/Switzerland/9715293/2013 (H3N2) antigen 0.015 MG/ML / influenza B virus B/Brisbane/9/2014 antigen 0.015 MG/ML Prefilled Syringe |
| [1794440] | 0.5 ML influenza A virus A/California/7/2009 (H1N1) antigen 0.03 MG/ML / influenza A virus A/Hong Kong/4801/2014 (H3N2) antigen 0.03 MG/ML / influenza B virus B/Brisbane/60 /2008 antigen 0.03 MG/ML / influenza B virus B/Phuket/3073/2013 antigen 0.03 MG/ML [1794448] influenza A virus A/California/7/2009 (H1N1) antigen 0.03 MG/ML / influenza A virus A/Hong Kong/4801/2014 (H3N2) antigen 0.03 MG/ML / influenza B virus B/Brisbane/60/2008 antigen 0.03 MG/ML / influenza B virus B/Phuket/3073/2013 antigen 0.03 MG/ML Injecta [1801070] 0.1 ML influenza A virus A/California/7/2009 (H1N1) antigen 0.09 MG/ML / influenza A virus A/Hong Kong/4801/2014 (H3N2) antigen 0.09 MG/ML / influenza B virus B/Brisbane/60 /2008 antigen 0.09 MG/ML / influenza B virus B/Phuket/3073/2013 antigen 0.09 MG/ML [1801161] 0.5 ML influenza A virus A/Christchurch/16/2010 (H1N1) antigen 0.03 MG/ML / influenza A virus A/Hong Kong/4801/2014 (H3N2) antigen 0.03 MG/ML / influenza B virus B/Brisban e/60/2008 antigen 0.03 MG/ML Prefilled Syringe |
| [1801178] | 0.5 ML influenza A virus A/California/7/2009 (H1N1) antigen 0.12 MG/ML / influenza A virus A/Hong Kong/4801/2014 (H3N2) antigen 0.12 MG/ML / influenza B virus B/Brisbane/60 /2008 antigen 0.12 MG/ML Prefilled Syringe |
| [1801187] | 0.5 ML influenza A virus A/California/7/2009 (H1N1) antigen 0.03 MG/ML / influenza A virus A/Hong Kong/4801/2014 (H3N2) antigen 0.03 MG/ML / influenza B virus B/Brisbane/60 /2008 antigen 0.03 MG/ML / influenza B virus B/Phuket/3073/2013 antigen 0.03 MG/ML [1801606] 0.5 ML influenza A virus A/Brisbane/10/2010 (H1N1) antigen 0.03 MG/ML / influenza A virus A/Hong Kong/4801/2014 (H3N2) antigen 0.03 MG/ML / influenza B virus B/Hong Kong/ 259/2010 antigen 0.03 MG/ML / influenza B virus B/Utah/9/2014 antigen 0.03 MG/ML Pref [1803019] 0.5 ML influenza A virus A/California/7/2009 (H1N1) antigen 0.09 MG/ML / influenza A virus A/Hong Kong/4801/2014 (H3N2) antigen 0.09 MG/ML / influenza B virus B/Brisbane/60 /2008 antigen 0.09 MG/ML Injection |
| [1928302] | 0.5 ML influenza A virus A/Hong Kong/4801/2014 (H3N2) antigen 0.03 MG/ML / influenza A virus A/Singapore/GP1908/2015 (H1N1) antigen 0.03 MG/ML / influenza B virus B/Bris bane/60/2008 antigen 0.03 MG/ML / influenza B virus B/Phuket/3073/2013 antigen 0.03 MG [1928330] 0.25 ML influenza A virus A/Hong Kong/4801/2014 (H3N2) antigen 0.03 MG/ML / influenza A virus A/Michigan/45/2015 (H1N1) antigen 0.03 MG/ML / influenza B virus B/Brisbane/ 60/2008 antigen 0.03 MG/ML / influenza B virus B/Phuket/3073/2013 antigen 0.03 MG/ML [1928339] 0.5 ML influenza A virus A/Hong Kong/4801/2014 (H3N2) antigen 0.12 MG/ML / influenza A virus A/Michigan/45/2015 (H1N1) antigen 0.12 MG/ML / influenza B virus B/Brisbane/6 0/2008 antigen 0.12 MG/ML Prefilled Syringe |
| [1928354] | influenza A virus A/Hong Kong/4801/2014 (H3N2) antigen 0.03 MG/ML / influenza A virus A/Michigan/45/2015 (H1N1) antigen 0.03 MG/ML / influenza B virus B/Brisbane/60/2008 antigen 0.03 MG/ML / influenza B virus B/Phuket/3073/2013 antigen 0.03 MG/ML Injectab |
| [1928360] | 0.5 ML influenza A virus A/Hong Kong/4801/2014 (H3N2) antigen 0.09 MG/ML / influenza A virus A/Michigan/45/2015 (H1N1) antigen 0.09 MG/ML / influenza B virus B/Brisbane/6 0/2008 antigen 0.09 MG/ML / influenza B virus B/Phuket/3073/2013 antigen 0.09 MG/ML P |
| [1928468] | 0.1 ML influenza A virus A/Hong Kong/4801/2014 (H3N2) antigen 0.09 MG/ML / influenza A virus A/Michigan/45/2015 (H1N1) antigen 0.09 MG/ML / influenza B virus B/Brisbane/6 0/2008 antigen 0.09 MG/ML / influenza B virus B/Phuket/3073/2013 antigen 0.09 MG/ML P |
| [1928960] | 0.5 ML influenza A virus A/Hong Kong/4801/2014 (H3N2) antigen 0.03 MG/ML / influenza A virus A/Singapore/GP1908/2015 (H1N1) antigen 0.03 MG/ML / influenza B virus B/Bris bane/60/2008 antigen 0.03 MG/ML Prefilled Syringe |
| [1928969] | influenza A virus A/Hong Kong/4801/2014 (H3N2) antigen 0.03 MG/ML / influenza A virus A/Singapore/GP1908/2015 (H1N1) antigen 0.03 MG/ML / influenza B virus B/Brisbane/60/ 2008 antigen 0.03 MG/ML Injectable Suspension |
| [1942127] | influenza virus vaccine 2017-2018 (trivalent generic for Afluria - Hong Kong, Singapore, Brisbane) 0.5 ML Prefilled Syringe |
| [1946984] | influenza virus vaccine, live attenuated, 2017-2018 Quadrivalent Nasal Spray [2048961] influenza virus vaccine 2018-2019 (quadrivalent - Singapore/Singapore/Maryland/Ph uket) 0.5 ML Prefilled Syringe |
| [2048969] | influenza virus vaccine 2018-2019 (quadrivalent - Singapore/Singapore/Maryland/Phuket) Injectable Suspension |
| [2050377] | influenza virus vaccine 2018-2019 (quadrivalent generic for Flublok - Michigan/Singa pore/Maryland/Phuket) 0.5 ML Prefilled Syringe |
| [2050418] | influenza virus vaccine 2018-2019 (trivalent - Michigan/Singapore/Maryland) 0.5 ML Prefilled Syringe |
| [2050758] | 0.5 ML influenza A virus A/Singapore/GP1908/2015 (H1N1) antigen 0.03 MG/ML / influenza A virus A/Singapore/INFIMH-16-0019/2016 (H3N2) antigen 0.03 MG/ML / influenza B virus B/Maryland/15/2016 antigen 0.03 MG/ML Prefilled Syringe |
| [2050766] | influenza virus vaccine 2018-2019 (trivalent - Singapore/Singapore/Maryland) Injectable Suspension |
| [2050772] | influenza virus vaccine 2018-2019 (quadrivalent - Michigan/Singapore/Maryland/Phuket) 0.5 ML Prefilled Syringe |
| [2050780] | 0.5 ML influenza A virus A/Michigan/45/2015 (H1N1) antigen 0.03 MG/ML / influenza A virus A/Singapore/INFIMH-16-0019/2016 (H3N2) antigen 0.03 MG/ML / influenza B virus B/ Maryland/15/2016 antigen 0.03 MG/ML / influenza B virus B/Phuket/3073/2013 antigen 0.03 M G/ML Injection |
| [2054272] | influenza A virus A/Singapore/INFIMH-16-0019/2016 (H3N2) antigen 158000000 U NT/ML / influenza A virus A/Slovenia/2903/2015 (H1N1) antigen 158000000 UNT/ML / influenza B virus B/Colorado/06/2017 antigen 158000000 UNT/ML / influenza B virus B/Phuket/3073/2 013 antigen 158000000 UNT/ML Nasal Spray |
| [2056528] | 0.5 ML influenza A virus A/North Carolina/04/2016 (H3N2) antigen 0.03 MG/ML / influenza A virus A/Singapore/GP1908/2015 (H1N1) antigen 0.03 MG/ML / influenza B virus B/Iowa/06/2017 antigen 0.03 MG/ML / influenza B virus B/Singapore/INFTT-16-0610/2016 antigen 0. 03 MG/ML Prefilled Syringe |
| [2056533] | influenza A virus A/North Carolina/04/2016 (H3N2) antigen 0.03 MG/ML / influenza A virus A/Singapore/GP1908/2015 (H1N1) antigen 0.03 MG/ML / influenza B virus B/Iowa/06/2 017 antigen 0.03 MG/ML / influenza B virus B/Singapore/INFTT-16-0610/2016 antigen 0.03 MG /ML Injectable Suspension |
| [2177393] | influenza virus vaccine 2019-2020 (quadrivalent - Brisbane/Kansas/Maryland/Phuket ) 0.5 mL Prefilled Syringe |
| [2177498] | influenza virus vaccine 2019-2020 (quadrivalent - Brisbane/Kansas/Maryland/Phuket ) 0.5 ML Injection |
| [2177503] | influenza virus vaccine 2019-2020 (quadrivalent - Brisbane/Kansas/Maryland/Phuket ) 0.25 ML Prefilled Syringe |
| [2177590] | influenza virus vaccine 2019-2020 (quadrivalent - Brisbane/Kansas/Maryland/Phuket ) Injectable Suspension |
| [2177692] | 0.5 ML influenza A virus A/Brisbane/02/2018 (H1N1) antigen 0.12 MG/ML / influenza A virus A/Kansas/14/2017 (H3N2) antigen 0.12 MG/ML / influenza B virus B/Maryland/15/2016 antigen 0.12 MG/ML Prefilled Syringe |
| [2178091] | 0.5 ML influenza A virus A/Brisbane/02/2018 (H1N1) antigen 0.09 MG/ML / influenza A virus A/Kansas/14/2017 (H3N2) antigen 0.09 MG/ML / influenza B virus B/Maryland/15/2016 antigen 0.09 MG/ML / influenza B virus B/Phuket/3073/2013 antigen 0.09 MG/ML Prefilled Syri nge |
| [2178357] | influenza virus vaccine 2019-2020 (generic for Fluad- Brisbane/Kansas/Maryland) 0.5 ML Prefilled Syringe |
| [235579] | trivalent influenza vaccine |
| [2379633] | 0.5 ML influenza A virus A/Guangdong-Maonan/SWL1536/2019 (H1N1) antigen 0.03 MG/ML / influenza A virus A/Hong Kong/2671/2019 (H3N2) antigen 0.03 MG/ML / influenza B virus B/Phuket/3073/2013 antigen 0.03 MG/ML / influenza B virus B/Washington/02/2019 antigen 0.03 MG/ML Prefilled Syringe |
| [2380586] | influenza virus vaccine 2020-2021 (quadrivalent - Guangdong-Maonan/Hong Kong/Phuket/Washington) 0.5 mL Injection |
| [2380595] | influenza virus vaccine 2020-2021 (quadrivalent - Hong Kong/Victoria/Phuket/Victori a) 0.5 ML Prefilled Syringe |
| [2380605] | influenza A virus A/Hong Kong/2671/2019 (H3N2) antigen 0.03 MG/ML / influenza A virus A/Victoria/2454/2019 (H1N1) antigen 0.03 MG/ML / influenza B virus B/Phuket/3073/201 3 antigen 0.03 MG/ML / influenza B virus B/Victoria/705/2018 antigen 0.03 MG/ML Injectable Suspension |
| [2380843] | influenza virus vaccine 2020-2021 (quadrivalent - Delaware/Nebraska/Darwin/Singa pore) Injectable Suspension |
| [2380849] | influenza virus vaccine 2020-2021 (quadrivalent - Delaware/Nebraska/Darwin/Singa pore) 0.5 ML Prefilled Syringe |
| [2380858] | influenza virus vaccine 2020-2021 (quadrivalent generic for Flublok - Hawaii/Minnesota/Phuket/Washington) 0.5 ML Prefilled Syringe |
| [2381145] | 0.5 ML influenza A virus A/Hong Kong/2671/2019 (H3N2) antigen 0.03 MG/ML / infl uenza A virus A/Victoria/2454/2019 (H1N1) antigen 0.03 MG/ML / influenza B virus B/Victoria/ 705/2018 antigen 0.03 MG/ML Prefilled Syringe |
| [2383546] | influenza virus vaccine 2020-2021 high-dose (quadrivalent - Guangdong-Maonan/Hong Kong/Phuket/Washington) 0.7 ML Prefilled Syringe |
| [477258] | Influenza Virus Trivalent-Split 2004 |
| [477266] | Influenza Virus Vaccine TV P-SURF 2004 |
| [477271] | Influenza Vaccine Trivalent 2004 (Live) |
| [477272] | Influenza Vaccine Trivalent 2004 (Live) Nasal Spray |
| [545244] | Influenza virus vaccine 0.015 MG/ML [Fluzone] |
| [5806] | Influenza virus vaccine |
| [857921] | Influenza Virus Vaccine, Inactivated B-Brisbane-60-2008 strain |
| [857965] | 0.25 ML Influenza Virus Vaccine, Inactivated A-Brisbane-59-2007, IVR-148 (H1N1) strain 0.03 MG/ML / Influenza Virus Vaccine, Inactivated A-Uruguay-716-2007, NYMC X-175C (H3 N2) strain 0.03 MG/ML / Influenza Virus Vaccine, Inactivated B-Brisbane-60-2008 str |
| [864701] | influenza A-California-7-2009-(H1N1)v-like virus vaccine |
| [895711] | Influenza Virus Vaccine, Live Attenuated, A-South Dakota-6-2007 (H1N1) (A-Brisbane- 59-2007-like) strain 158000000 UNT/ML / Influenza Virus Vaccine, Live Attenuated, A-Uruguay -716-2007 (H3N2) (A-Brisbane-10-2007-like) strain 158000000 UNT/ML / Influenza V |

**Table S3. Baseline Demographic and Clinical Characteristics of the COVID-19 and Flu Vaccine Cohorts with No History of COVID-19 Infection (Sensitivity Analysis), Before and After hdPS Matching**

|  | **BEFORE MATCHING** | | | **AFTER MATCHING** | | |
| --- | --- | --- | --- | --- | --- | --- |
|  | **FluVax**  **N=40390** | **COVIDVax**  **N=25556** | **SMD** | **FluVax**  **N=17530** | **COVIDVax**  **N=17530** | **SMD** |
| **Demographics** |  |  |  |  |  |  |
| Female, n (%) | 21870 (54.1%) | 13992 (54.8%) | 0.0121 | 9449 (53.9%) | 9533 (54.4%) | 0.0096 |
| Mean age, years (SD) | 62 (9) | 68.4 (9.2) | 0.7043 | 66.3 (9.5) | 66.0 (8.9) | 0.0257 |
| Age range, n (%) |  |  |  |  |  |  |
| 50-59 years | 19533 (48.4%) | 5193 (20.3%) | 0.6038 | 4726 (27%) | 4819 (27.5%) | 0.0119 |
| 60-69 years | 13444 (33.3%) | 8703 (34.1%) | 0.0163 | 7202 (41.1%) | 6803 (38.8%) | 0.0465 |
| 70-79 years | 5271 (13.1%) | 8972 (35.1%) | 0.5553 | 3813 (21.8%) | 4865 (27.8%) | 0.1394 |
| 80-89 years | 2142 (5.3%) | 2688 (10.5%) | 0.2011 | 1789 (10.2%) | 1043 (5.9%) | 0.1567 |
| Race, n (%) |  |  |  |  |  |  |
| White | 24277 (60.1%) | 15577 (61%) | 0.0173 | 10862 (62%) | 10470 (59.7%) | 0.0458 |
| Asian | 7364 (18.2%) | 5549 (21.7%) | 0.0878 | 3106 (17.7%) | 3863 (22%) | 0.1084 |
| Other | 6539 (16.2%) | 3606 (14.1%) | 0.0577 | 2614 (14.9%) | 2646 (15.1%) | 0.0051 |
| Black | 2210 (5.5%) | 824 (3.2%) | 0.1074 | 948 (5.4%) | 551 (3.1%) | 0.1121 |
| Hispanic ethnicity, n (%) | 3439 (8.5%) | 1452 (5.7%) | 0.1083 | 1346 (7.7%) | 1049 (6%) | 0.0672 |
| **History in EHR** |  |  |  |  |  |  |
| Mean pre-index days (SD) | 3060 (2161.4) | 4196.7 (2567.9) | 0.4884 | 3136.9 (2218.2) | 4146 (2566.8) | 0.4207 |
| Mean follow-up days (SD) | 1336.5 (564.4) | 393.8 (214.8) | 2.0427 | 1303.4 (573.7) | 385.4 (213.1) | 2.1213 |
| Mean encounters (SD) | 19.7 (34.5) | 9 (22) | 0.3519 | 10.1 (24.7) | 9.9 (24.2) | 0.0051 |
| **Comorbidities** |  |  |  |  |  |  |
| Mean CCI score (SD) | 3.1 (2.6) | 4 (2.8) | 0.3570 | 3.6 (2.7) | 3.5 (2.6) | 0.0387 |
| CCI comorbidities, n (%) |  |  |  |  |  |  |
| Malignancy | 4649 (11.51%) | 4720 (18.47%) | 0.2003 | 2363 (13.48%) | 2776 (15.84%) | 0.0666 |
| Metastatic solid tumor | 1076 (2.66%) | 1012 (3.96%) | 0.0741 | 526 (3%) | 547 (3.12%) | 0.0070 |
| Diabetes | 6983 (17.29%) | 4019 (15.73%) | 0.0419 | 3105 (17.71%) | 2487 (14.19%) | 0.0964 |
| Diabetes with complications | 2542 (6.29%) | 1626 (6.36%) | 0.0028 | 1245 (7.1%) | 943 (5.38%) | 0.0713 |
| Congestive heart failure | 1906 (4.72%) | 1896 (7.42%) | 0.1160 | 1007 (5.74%) | 1017 (5.8%) | 0.0024 |
| Myocardial infarction | 990 (2.45%) | 1051 (4.11%) | 0.0960 | 504 (2.88%) | 602 (3.43%) | 0.0320 |
| Peripheral vascular disease | 1736 (4.3%) | 1944 (7.61%) | 0.1445 | 954 (5.44%) | 1086 (6.2%) | 0.0322 |
| Chronic pulmonary disease | 6552 (16.22%) | 3712 (14.52%) | 0.0468 | 2846 (16.24%) | 2336 (13.33%) | 0.0820 |
| Cerebrovascular disease | 2038 (5.05%) | 1992 (7.79%) | 0.1149 | 1121 (6.39%) | 1068 (6.09%) | 0.0125 |
| Dementia | 442 (1.09%) | 357 (1.40%) | 0.0277 | 324 (1.85%) | 157 (0.90%) | 0.0820 |
| Hemiparaplegia | 313 (0.77%) | 219 (0.86%) | 0.0092 | 136 (0.78%) | 126 (0.72%) | 0.0066 |
| Mild liver disease | 2852 (7.06%) | 2323 (9.09%) | 0.0755 | 1199 (6.84%) | 1498 (8.55%) | 0.0640 |
| Severe liver disease | 289 (0.72%) | 182 (0.71%) | 0.0004 | 127 (0.72%) | 92 (0.52%) | 0.0253 |
| Renal disease | 2929 (7.25%) | 2520 (9.86%) | 0.0949 | 1619 (9.24%) | 1396 (7.96%) | 0.0454 |
| Peptic ulcer disease | 548 (1.36%) | 476 (1.86%) | 0.0409 | 264 (1.51%) | 286 (1.63%) | 0.0101 |
| Rheumatic disease | 1204 (2.98%) | 880 (3.44%) | 0.0264 | 580 (3.31%) | 549 (3.13%) | 0.0100 |
| HIV | 149 (0.37%) | 44 (0.17%) | 0.0364 | 59 (0.34%) | 38 (0.22%) | 0.0228 |

Caption: Blue indicates SMD ≥0.1 and that the characteristic is unbalanced between the cohorts. Abbreviations: CCI, Charlson Comorbidity Index; EHR, electronic health records; HIV, human immunodeficiency virus; hdPS, high-dimensional propensity score; SD, standard deviation; SMD, standardized mean difference.
